# High Prevalence of *hefA* Efflux Pump Overexpression in Isolates of *Helicobacter pylori* Resistant to Clarithromycin

**DOI:** 10.3390/antibiotics14101044

**Published:** 2025-10-18

**Authors:** Marcela Villegas, Catalina Ortega, Krishna Gómez, Alvaro Cerda, Rolando Sepúlveda, Christian Lara, Luis Bustamante, Daniela Garcia, Luis Coppelli, Edmundo Hofmann, Armando Sierralta, Mónica Pavez

**Affiliations:** 1 Center of Excellence in Translational Medicine, CEMT-BIOREN, Universidad de La Frontera, Temuco 4810296, Chile; m.villegas06@ufromail.cl (M.V.);; 2 Doctoral Program in Sciences with a Mention in Applied Cellular and Molecular Biology, Universidad de La Frontera, Temuco 4811230, Chile; 3 Department of Basic Sciences, Universidad de La Frontera, Temuco 4811230, Chile; 4 Department of Internal Medicine, Universidad de La Frontera, Temuco 4781218, Chile; 5 Gastroenterology Unit, Hospital Hernan Henriquez Aravena, Temuco 4781151, Chile; 6 Gastroenterology Unit, Hospital de Villarrica, Villarrica 4930000, Chile; 7 Gastroenterology Unit, Clinica Alemana de Temuco, Temuco 4810297, Chile

**Keywords:** *Helicobacter pylori*, clarithromycin resistance, *hefA* efflux pump overexpression, *23S rRNA* mutations

## Abstract

**Background/Objectives: ***Helicobacter pylori* is a cause of chronic gastric infections and gastrointestinal carcinogenesis, with a prevalence of 20–90% around the world. Its eradication is increasingly challenged by clarithromycin resistance, particularly in regions with high rates of antibiotic resistance. While clarithromycin resistance is primarily attributed to *23S rRNA* mutations, secondary mechanisms such as efflux pumps remain understudied. The present study reports a high prevalence of *hefA* efflux pump overexpression as a main molecular basis of clarithromycin resistance in *H. pylori* isolates from southern Chile. **Materials and Methods:** A total of 102 *H. pylori* isolates were obtained from gastric biopsy cultures. Isolates were analyzed for clarithromycin susceptibility by MIC, the *23S rRNA* mutations A2142G/A2143G by PCR-RFLP followed by sequencing, and *hefA* relative expression by qPCR. **Results:** Clarithromycin resistance was detected in 38% of isolates. Resistance was significantly associated with therapeutic failure and urban residence. While 44% of resistant isolates harbored A2142G/A2143G mutations, 56% did not, suggesting alternative resistance mechanisms. Mutation C2182T was identified in 11% resistant isolates, and increased *hefA* expression was observed in resistant strains without *23S rRNA* mutations, indicating efflux pump dysregulation as a resistance mechanism. **Conclusions:** Our findings reveal a shift in epidemiology of clarithromycin resistance mechanisms in *H. pylori* that extends beyond classical *23S rRNA* mutations to include efflux-mediated adaptive resistance as a contributing mechanism. The positive correlation between *hefA* overexpression and MIC elevation underscores its role in resistance. These findings have important implications for the efficacy of clarithromycin-based therapies and highlight the need to reassess empirical treatment strategies in response to emerging resistance patterns.

## 1. Introduction

*Helicobacter pylori* is a chronic infection that has become prevalent around the world, colonizing more than half of humanity [1]. Its prevalence is approximately 50% in developed countries and up to 90% in underdeveloped regions [2]. The presence of this bacterium is a critical factor in the carcinogenesis process for most patients who develop differentiated gastric adenocarcinoma, progressing through stages of chronic gastritis, atrophy, and metaplastic changes [3]. Consequently, the eradication of *H. pylori* is considered a public health priority at both global and national levels.

Antibiotic resistance is a key determinant in the failure of eradication treatments and the recurrence of *H. pylori* infections. Numerous susceptibility studies have assessed treatment efficacy in different regions, particularly concerning clarithromycin resistance [4]. In cases where resistance is present, therapeutic success rates can drop to 40% or lower. A meta-analysis conducted by Fischbach and Evans reported a 66.2% reduction in eradication rates when the bacterium exhibited resistance to clarithromycin [5]. Other studies determined that the eradication rate of *H. pylori* in clarithromycin-resistant strains is 32% lower than in sensitive strains. In general, regions with high antibiotic resistance face significantly reduced eradication rates with empirical treatments for *H. pylori*, unlike pre-treatment susceptibility studies, which show higher eradication rates, with differences up to 20% to 50% in effectiveness [6]. Factors such as antibiotic resistance and patient demographics likely contribute to these disparities. Notably, current treatment guidelines do not incorporate susceptibility testing for managing the infection, nor do they address a comprehensive understanding of resistance mechanisms.

Clarithromycin resistance is the most extensively studied mechanism contributing to *H. pylori* eradication failure [4]. Specific mutations inhibit clarithromycin’s action by preventing its binding to the ribosomal subunit responsible for protein synthesis [7]. Three primary mutations of 23S rRNA gene—A2142G, A2143G, and A2142C—are the main contributors to clarithromycin resistance in *H. pylori*. Each mutation confers varying levels of resistance, with reports of A2143G causing the most significant reduction in eradication rates (30.7%) and greatly impacting treatment outcomes [8]. Interestingly, mutations at position 2142 result in higher resistance levels (MIC > 64 µg/mL) compared to those at position 2143 [9]. Although 90% of clarithromycin resistance is attributed to these three mutations, other mutations have been identified, though their role in resistance remains controversial and lacks clinical validation [10]. A secondary mechanism associated with clarithromycin resistance involves efflux pumps, which reduce the intracellular concentration of the antibiotic [10]. This mechanism may or may not act synergistically with *23S rRNA* mutations to exacerbate resistance. A study of 15 clarithromycin-resistant *H. pylori* strains revealed the expression of a Resistance-nodulation-division (RND) efflux pump, with a significant reduction in MIC values when pump inhibitors were applied [10]. Despite its relevance to clarithromycin resistance, this mechanism is less studied due to the complexity of phenotypic and molecular analyses and the limited availability of clinical evidence.

Global studies on clarithromycin susceptibility report resistance rates ranging from 15% in Sweden to 96% in Australia and 43% in Chile, with mean interregional variations in resistance exceeding 20% [11,12]. However, the molecular mechanisms driving resistance have not been comprehensively studied, especially in Latin American countries.

In this context, this study reports a potential epidemiological shift in the predominant mechanism of clarithromycin resistance in *H. pylori* isolates from dyspeptic patients in southern Chile, where the efflux mechanism was found to predominate over target-site mutations. This finding could redefine current perspectives on resistance pathways and inform more effective treatment and surveillance strategies.

## 2. Results

### 2.1. Clarithromycin Susceptibility

Resistance to clarithromycin (MIC > 0.25 μg/mL) was detected in 35.3% of the 102 *H. pylori* isolates analyzed, using a MIC50 of 0.125 μg/mL and a MIC90 of >32 μg/mL. The MIC range for clarithromycin susceptibility varied from <0.016 to >256 μg/mL. Sociodemographic analysis revealed a higher prevalence of clarithromycin-resistant *H. pylori* isolates in patients living in urban areas (Table 1) with individuals being four times more likely to harbor a resistant strain compared to those living in rural areas (OR: 4.0000, IC: 1.1794–13.5657). Also, a statistically significant association was observed with therapeutic failure in these patients, demonstrating a 6.4-fold increased risk of clarithromycin resistance in patients previously exposed to the antibiotic (OR: 6.4000, IC (1.9356–21.1618).

### 2.2. 23S rRNA Mutations (A2142G, A2143G)

**Figure 1:**
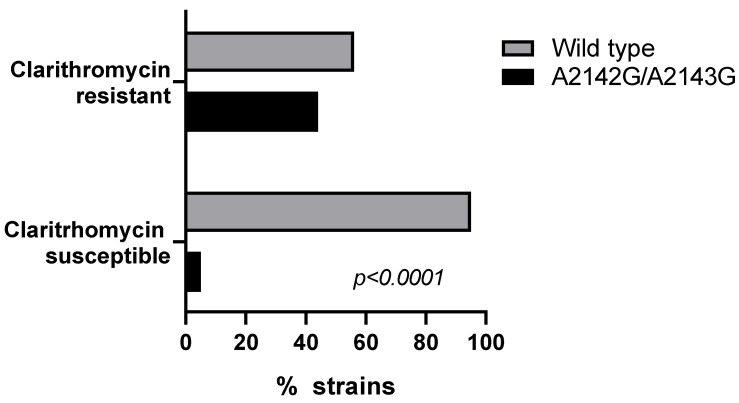
Association between the presence of A2142G and/or A2143G mutations of 23S rRNA and clarithromycin susceptibility profile in *H. pylori* strains. Data represent the frequencies of wild-type or A2142G/A2143G mutant strains in Clarithromycin-resistant and Clarithromycin-susceptible isolates. Data are presented as percentage and were compared using chi-square test.

Genotyping of the *23S rRNA* gene by PCR-RFLP was conducted on 86 isolates to identify the A2142G and A2143G mutations, including 27 clarithromycin-resistant and 59 clarithromycin-sensitive isolates. While the frequency of these mutations was significantly higher in clarithromycin-resistant as compared to clarithromycin-susceptible strains (44% vs. 5%; *p* < 0.0001), it is interesting that only 44% (n = 12) of the resistant isolates harbored one of these mutations (Figure 1). Specifically, 11 isolates (40.7%) exhibited the A2143G mutation, and one isolate (3.7%) exhibited the A2142G mutation. Interestingly, 5% (three clarithromycin-susceptible isolates) also showed the presence of these mutations, with one isolate (1.7%) harboring A2142G and two isolates (3.3%) harboring A2143G (Figure 1).

Notably, 55.6% (n = 15) of the clarithromycin-resistant strains did not exhibit either of the initially studied mutations. Sequencing of the V domain of the *23S rRNA* gene in these isolates revealed eight-point mutations (A1826G, C1707T, C1822T, C1830T, G1821A, T1916G, T1916C, C2182T). However, when comparing the sequences of resistant and sensitive strains, only one mutation (C2182T) was consistently associated with clarithromycin resistance, as observed in three isolates (Hp187 MIC = 4 μg/mL, Hp309 MIC = 32 μg/mL, Hp575 MIC ≥ 256 μg/mL).

### 2.3. hefA Gene Expression

**Figure 2:**
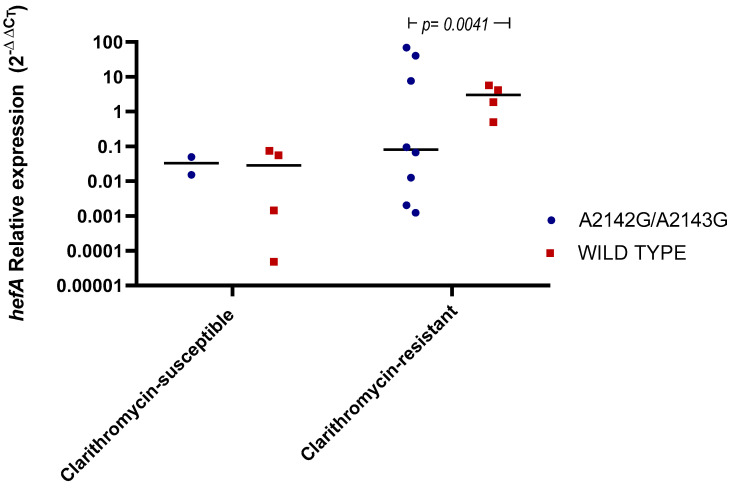
Relative expression of *hefA* in *H. pylori* strains classified as either clarithromycin-susceptible or clarithromycin-resistant, with and without A2142G/A2143G mutations. Data represent dispersion graph with bars indicating median values. The Mann–Whitney U test was used to compare values between clarithromycin-susceptible and resistant and between wild-type and A2142G/A2143G mutant strains into each group.

To investigate potential efflux mechanisms contributing to clarithromycin resistance, the transcriptional levels of the *hefA* gene were analyzed across different MIC levels of the bacterial isolates, covering the full range of observed susceptibility. A total of 19 isolates were quantified preserving the A2141G and A2143G mutation frequencies observed in the resistant cohort. Although no significant differences were observed in *hefA* expression between clarithromycin-sensitive and -resistant *H. pylori* groups, a significant increase in *hefA* mRNA expression was detected in a subset of resistant strains without *23S rRNA* mutations (*p* = 0.041) (Figure 2). This suggests a dysregulation of the efflux system in bacteria lacking classical mutations of *23S rRNA*.

**Figure 3:**
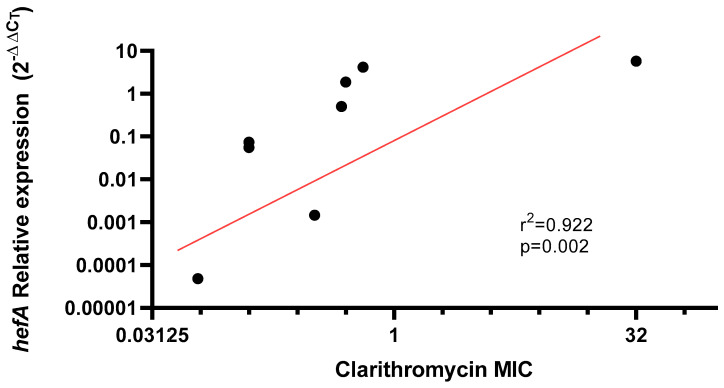
Association between Clarithromycin minimum inhibitory concentration (MIC) values and *hefA* gene expression in strains without *23S rRNA* mutation.

Furthermore, a positive correlation (*R* = 0.92) was observed between *hefA* relative expression in strains without *23S rRNA* mutations and clarithromycin MIC levels, indicating that *hefA* overexpression is associated with increased resistance in the absence of mutations (*p* = 0.002) (Figure 3).

## 3. Discussion

The increasing resistance to clarithromycin in *H. pylori* is a major concern highlighted in the Maastricht VI/Florence Consensus Report [13]. Our study observed a resistance rate of 38%, comparable to rates of 26–31% reported in Chile [14,15] and consistent with global trends exceeding 15% in most WHO regions [16]. Given that resistance rates above 20% are associated with a 30–60% reduction in eradication success [17]. This pattern is evident in the population studied, where one-third of patients harboring clarithromycin-resistant strains experienced treatment failure (Table 1). Our findings demonstrate a 6.8-fold increased risk of clarithromycin resistance among patients with prior exposure to the antibiotic treatment, underscoring the importance of considering antibiotic history when selecting treatment regimens for *Helicobacter pylori* infection. Other authors corroborated the decrease in eradication rates when resistance rates surpassed 20% [15]. Local studies associated with our research group have reported a decline in eradication rates in recent years, prompting the need to reformulate empirical therapies [18].

From a demographic perspective, our findings highlight population characteristics associated with resistance to clarithromycin (Table 1), notably, a higher prevalence of resistant isolates in urban areas. These findings revealed a higher prevalence of resistant isolates in urban areas, where individuals were four times more likely to harbor resistant *H. pylori* strains. This disparity may reflect greater antibiotic exposure and environmental selective pressure, supported by studies showing higher macrolide resistance and abundance of resistance genes in urban wastewater [19,20].

Macrolide resistance in *H. pylori* has been predominantly attributed to point mutations in the *23S rRNA* gene at positions 2142 and 2143 (A2142G, A2143G, A2142C) [21]. A secondary mechanism involving efflux pump systems has been less studied and reported with low prevalence [10,22]. In this context, our study provides new molecular insights into clarithromycin resistance in clinical *H. pylori* isolates, revealing a higher prevalence of this underlying mechanism.

The *23S rRNA* point mutations were observed in only 45% of clarithromycin-resistant isolates, with the majority associated with A2142G, A2143G, and 3 strains with T2182C mutation [23]. Notably, 55% of resistant isolates without mutations exhibited possible adaptive resistance involving overexpression of the efflux pump system HefABC (Figure 1 and Figure 2). This predominance of the efflux mechanism over target-site mutations could represent a potential epidemiological shift in the predominant resistance mechanism of clarithromycin in *H. pylori* at the global level, now observed in the southern Chilean population studied.

The *hefA* gene has been associated with enhanced antibiotic resistance and biofilm formation, both reducing *H. pylori* susceptibility to macrolides [24]. Experimental data confirm that efflux pump inhibition can restore clarithromycin susceptibility, yet clinical evidence remains scarce. This is the first time a positive correlation has been described between efflux pump gene expression of clinical isolates and an increase in the MIC for clarithromycin (r = 0.92), suggesting the rise in resistance related to the dysregulation of the efflux pump system (Figure 3). This behavior is observed in various Gram-Negative bacilli exposed to macrolide, β-lactam, and quinolone antibiotics [25,26]. Studies on Gram-Negative strains have shown that membrane impermeability is an SOS response to the selective pressure exerted by these antibiotics. However, these mechanisms significantly impact bacterial fitness due to the nonspecific nature of efflux pumps. When overexpressed, these pumps expel various molecules that may be essential for bacterial survival [27]. This is why the need for definitive adaptation, such as the presence of mutations, allows bacteria to achieve greater survival stability, rather than relying solely on impermeability-based survival mechanisms [26,28]. This is consistent with our results, where no correlation was observed between *hefA* relative expression and MIC in A2142G/A2143G mutated isolates, showing a wide variation in *hefA* expression levels (Figure 2). It is important to mention that HefA efflux pump has been linked to the reduction in intracellular concentrations of various structurally unrelated drugs, bile salts, and metal ions, meaning it would not act as a specific antimicrobial mechanism in *H. pylori* [29].

The overexpression of *hefA* as a resistance mechanism to clarithromycin, in the absence of A2142G/A2143G mutations in clinical isolates of *H. pylori* from southern Chile, could be triggered by phenotypic instability due to constant exposure. Bacteria may continue to overexpress efflux pumps even in the absence of the stimulating drug [30]. This is why the high phenotypic resistance rates observed in this region, may be linked to regulatory mechanisms of membrane impermeability due to prior exposure to these compounds.

Chile is one of the countries with the highest per capita macrolide consumption in the region [31]. This prolonged exposure could promote overexpression of efflux pumps in gastric isolates. Further comprehensive studies are needed to fully understand the dynamics of this mechanism in *H. pylori*.

Our findings strongly suggest that these resistance mechanisms are influencing the bacterium’s susceptibility to clarithromycin, where 56% of resistant strains were not linked to classical mutations (A2142G and A2143G) of *23S rRNA* gene. This perspective on clarithromycin resistance in clinical isolates observed in our population could have significant implications for surveillance strategies and therapeutic decision-making, especially if susceptibility studies are limited to genetic studies of classical mutations. In populations with poorly regulated antibiotic use, the selective pressure of antibiotic exposure poses a real risk to the development of emerging resistance mechanisms.

The main limitation of our study is the absence of phenotypic validation of efflux pump activity (e.g., MIC determination with RND inhibitors or EtBr accumulation/efflux assays). Thus, transcriptional upregulation is interpreted as suggestive of contributing mechanisms. Further molecular and functional research linking this mechanism to treatment failure is necessary to confirm its clinical significance and optimize treatment strategies, particularly considering that this predominance of the efflux mechanism over target-site mutations represents a potential epidemiological shift in the predominant resistance mechanism of clarithromycin in *H. pylori* at the global level, now observed in the southern Chilean population studied.

## 4. Materials and Methods

### 4.1. Patients Recruitment and Obtention of Biological Samples

A total of 102 *H. pylori* isolates were obtained from gastric biopsy cultures of adult dyspeptic patients undergoing endoscopy at three major health institutions in the La Araucanía Region of southern Chile. Patients were included in the study after signing the informed consent previously approved by the Bioethical Committee of Universidad de La Frontera, Chile (Number No. 102/2019). All patients were over 18 years old, and they had not taken antibiotics and proton pump inhibitors within three weeks prior to endoscopy. Before sample collection, individuals were questioned about their sociodemographic characteristics, such as their highest level of education, main occupation, gender, age, race, Mapuche ethnicity, and location of residence. Questions about clinical history included current medications, history of gastric cancer, history of *H. pylori* infection, and treatments for the bacteria to evaluate previous management of *H. pylori*. All recruitment and biological samples obtentions were carried out in accordance with the Declaration of Helsinki statements.

### 4.2. Culture and Phenotypic Identification of H. pylori

Biopsy samples were macerated, homogenized, and inoculated onto Columbia blood agar (DIFCO, Franklin Lakes, NJ, USA) supplemented with 7% horse blood and selective antibiotics for *H. pylori*, including trimethoprim, amphotericin B, polymyxin B (5 µg/mL each), and vancomycin (10 µg/mL) (DENT Supplement, Thermo. Scientific™ Oxoid™, Hampshire, UK). Cultures were incubated for 5–10 days at 37 °C under microaerophilic conditions (5% O_2_, 10% CO_2_, 85% N_2_, and 90% humidity) using commercial systems (Campypack, Thermo. Scientific™ Oxoid™, Hampshire, UK). Growth suggestive of *H. pylori* was confirmed through phenotypic tests, including oxidase, catalase, and Gram staining to assess bacterial morphology. Presumptive *H. pylori* isolates were suspended in Columbia broth with 5% horse serum and 20% glycerol and stored at −80 °C until use.

### 4.3. Antibiotic Susceptibility Testing

Antibiotic susceptibility was evaluated using the epsilometry method (E-test, Thermo. Scientific™ Oxoid™, Hampshire, UK) to determine the minimum inhibitory concentration (MIC) of clarithromycin, following the European Committee on Antimicrobial Susceptibility Testing (EUCAST) guidelines [32]. Fresh colonies were suspended in sterile distilled water to achieve a standard bacterial concentration of 3 McFarland (9.0 × 10^8^ CFU/mL). MIC values were interpreted using clinical breakpoints for clarithromycin resistance (>0.25 µg/mL). *H. pylori* ATCC 43504 was used as a quality control strain.

### 4.4. DNA Extraction from Pure Culture

DNA was extracted from isolated strains using the UltraClean^®^ Microbial DNA Isolation Kit (QIAGEN, Hilden, Germany), following the manufacturer’s instructions. Extracted DNA was stored at −20 °C.

### 4.5. Molecular Confirmation of H. pylori

Extracted DNA was used for species confirmation by PCR amplification of the *ureC* gene using the primers FExt: 5′-AGCTATAAAGTGGGCGAGAG-3′ and RExt: 5′-ATTGCCCGTTAGGCTCAT-3′. PCR amplifications were conducted under conventional conditions as described by researchers [33]. Amplified products were visualized using 1% agarose gel electrophoresis.

### 4.6. RFLP Analysis for A2142G and A2143G Mutations

A conventional PCR was performed to detect the V domain of the *23S rRNA* gene using primers F: 5′-AGATTGGAGGGAAGGCAAAT-3′ and R: 5′-CTCCATAAGAGCCAAAGCCC-3′. Amplification conditions included one cycle of denaturation at 94 °C for 5 min, followed by 35 cycles of denaturation at 94 °C for 30 s, annealing at 55 °C for 30 s, and extension at 72 °C for 30 s, with a final extension at 72 °C for 5 min. PCR products were visualized on 1% agarose gel electrophoresis. Restriction fragment length polymorphism (RFLP) analysis was conducted using BbsI (New England Biolabs, Ipswich, MA, USA) and BsaI (New England Biolabs, Ipswich, MA, USA) to study A2142G and A2143G mutations, respectively, as predicted by NEBcutter V2.0 (New England Biolabs, Ipswich, MA, USA). PCR products were incubated with restriction enzymes following the manufacturer’s instructions, and fragments were visualized on 1% agarose gel.

### 4.7. 23S rRNA Gene Sequencing

For strains with no mutations detected by RFLP, the V region of the *23S rRNA* gene was sequenced to identify other potential mutations. PCR products were purified using the Gel Band Purification Kit (GE Healthcare Life Sciences, Chicago, IL, USA) and sequenced at Macrogen Korea^®^ (Seoul, Republic of Korea). Sequencing data were analyzed with MEGA 11.0 software and compared to the NCBI database.

### 4.8. hefA Gene Relative Expression

Total RNA was extracted from cultures in exponential growth using the E.Z.N.A.^®^ Total RNA Kit I (OMEGA Bio-tek, Norcross, GA, USA). RNA quantity and purity were assessed using the QuantiFluor RNA System (Promega, Madison, WI, USA). RNA samples were treated with DNase (Ambion, Thermo Scientific™, Waltham, MA, USA) and reverse transcribed using Superscript II reverse transcriptase (Invitrogen, Carlsbad, CA, USA). Quantitative PCR was performed using the Applied Biosystems™ 7500 Fast Dx Real-Time PCR System and SYBR Green PCR Master Mix (Applied Biosystems, Thermo. Scientific™, Waltham, MA, USA). The *16S rRNA* gene was used as a housekeeping gene with primers F: 5′-GCGGGATAGTCAGTCAGGTCAGGTG-3′ and R: 5′-CATCGTTTAGGGCGTGGACT-3′. *hefA* relative expression was assessed using primers designed for this study (F: 5′-CTCGCTCGCATGATCGC-3′ and R: 5′-CGTATTCGCTCAAATTCCCT-3′). PCR conditions included one cycle at 50 °C for 2 min, one cycle at 95 °C for 2 min, 40 cycles at 95 °C for 15 s, and a final step at 60 °C for 1 min. Dissociation curves confirmed amplification specificity. Relative mRNA expression was calculated using the 2^−ΔΔCt^ method with non-induced isolates as the calibrator [34].

### 4.9. Statistical Analysis

Statistical analyses were performed in the program Minitab^®^18 and GraphPad Prism 8.0. The chi-square test of association was used to establish differences between the sociodemographic characteristics with an established degree of significance of *p* < 0.05. To determine variables associated with the risk of harboring a Clarithromycin-resistant *H. pylori* infection, binary logistic regression models were adjusted, obtaining the odds ratio (OR) and its 95% confidence intervals. Clarithromycin susceptibility and prevalence data were analyzed descriptively to determine MIC_50_ and MIC_90_ values, and the presence of A2142G and A2143G mutations was associated with antibiotic resistance using Fisher’s exact test. *hefA* gene relative expression differences between resistant and sensitive, as well as mutated and non-mutated, strains were analyzed using Mann–Whitney and *t*-tests. Statistical analyses were performed using Prism software, with significance set at *p* < 0.05.

## 5. Conclusions

This study highlights the evolving landscape of clarithromycin resistance in *H. pylori* isolated in southern Chile. The resistance rate observed in our population aligns with global trends, where the emergence of alternative resistance mechanisms, such as efflux pump overexpression, is becoming increasingly prevalent. The majority of resistant isolates in our study did not exhibit classical *23S rRNA* mutations but instead demonstrated adaptive gene expression of impermeability membrane mechanisms, particularly involving the HefABC efflux pump system. These findings suggest that relying solely on genetic studies of classical mutations may underestimate the complexity of resistance in *H. pylori*, highlighting the need for broader approaches in susceptibility testing.

The impact of prolonged antibiotic exposure could be related to our findings, where efflux pump overexpression may be driven by selective pressure, particularly in urban regions with high antibiotic consumption. This has significant implications for the efficacy of clarithromycin-based therapies and underscores the importance of revisiting empirical treatment strategies in light of emerging resistance patterns. While this study provides valuable insights into the resistance mechanisms of *H. pylori*, further research is needed to explore the clinical significance and functional analysis of efflux pump-mediated resistance.

## Figures and Tables

**Table 1:** Sociodemographic characteristics of patients according to clarithromycin susceptibility profile against *H. pylori*.

Background	Patients with Clarithromycin Resistant Strains n = 36	Patients with Clarithromycin Sensitive Strains n = 66	*p* Value
Age (years)	47.2 ± 13.7	49.2 ± 12.2	0.466
Sex (female)	20 (55.56%)	49 (74.24%)	0.054
Urban residence	32 (88.9%)	43 (64.7%)	0.012 *^ƚ^
Education level ≤ 12 years	20 (57.1%)	46 (74.2%)	0.084
Public health insurance	28 (80%)	60 (92.3%)	0.071
Family group ≥ 5 members	6 (17.1%)	7 (10.8%)	0.366
Smoker	7 (19.4%)	24 (36.9%)	0.068
Drinker	17 (47.2%)	35(53.8%)	0.524
Previous treatment for *H. pylori*	12 (33.3%)	5 (7.6%)	0.001 *^ƚƚ^

* statistically significant; ^ƚ^ OR: 4.0000, IC: 1.1794–13.5657; ^ƚƚ^ OR: 6.4000, IC: 1.9356–21.1618.

## Data Availability

Sequence repository ID: PV156774, PV156775, PV156776, PV156777, PV156778, PV156779 Link: https://www.ncbi.nlm.nih.gov/nuccore (accessed on: 28 February 2025).

## Abbreviations

The following abbreviations are used in this manuscript:

RFLP	Restriction fragment length polymorphism
WHO	World Health Organization
ARG	antibiotic resistance genes
*H. pylori*	*Helicobacter pylori*
